# Mito-Nuclear Communication in Hepatocellular Carcinoma Metabolic Rewiring

**DOI:** 10.3390/cells8050417

**Published:** 2019-05-05

**Authors:** Tommaso Mello, Irene Simeone, Andrea Galli

**Affiliations:** 1 Clinical Gastroenterology Unit, Department of Biomedical Clinical and Experimental Sciences “Mario Serio”, University of Florence, V.le Pieraccini 6, Florence 50129, Italy; irene.simeone@student.unisi.it (I.S.); andrea.galli@unifi.it (A.G.); 2 University of Siena, 53100 Siena, Italy

**Keywords:** HCC, PPAR, SIRT, PGC-1, NRF, HIF, liver, mitochondria, metabolism

## Abstract

As the main metabolic and detoxification organ, the liver constantly adapts its activity to fulfill the energy requirements of the whole body. Despite the remarkable adaptive capacity of the liver, prolonged exposure to noxious stimuli such as alcohol, viruses and metabolic disorders results in the development of chronic liver disease that can progress to hepatocellular carcinoma (HCC), which is currently the second leading cause of cancer-related death worldwide. Metabolic rewiring is a common feature of cancers, including HCC. Altered mito-nuclear communication is emerging as a driving force in the metabolic reprogramming of cancer cells, affecting all aspects of cancer biology from neoplastic transformation to acquired drug resistance. Here, we explore relevant aspects (and discuss recent findings) of mito-nuclear crosstalk in the metabolic reprogramming of hepatocellular carcinoma.

## 1. Introduction

A tumor is a very harsh environment to live in. Poor oxygenation, low nutrient levels, high concentration of waste metabolites, and acidic pH are inevitable consequences of a crowded and disorganized mass of fast-growing cells. Moreover, the tumor microenvironment can change dramatically within the growing mass, because of the defective tumor vasculature, necrosis, immune response and therapeutic treatments. This environment operates an enormous selective pressure that, combined with the poor genomic stability of cancer cells, leads to cancer cell evolution and the acquisition of a progressively malignant phenotype. An early-enabled characteristic of the malignant transformation of cancer cells is the reprogramming of their energy metabolism in order to support the cell fast growing rate. It has been long noted that cancer cells rely primarily on glycolysis for adenosine triphosphate (ATP) production, even in the presence of oxygen (Warburg effect) [1]. However, only more recently the significance of this “metabolic reprogramming”, its plasticity, its implications in cancer biology and response to treatment have begun to emerge [2]. Otto Warburg proposed that “aerobic glycolysis” was due to defective mitochondria respiration that forces cancer cells to rely on an alternative pathway for energy production [3]; it is now clear that mitochondria are not simply dysfunctional in cancer cells. Rather, they are reprogrammed to serve as “biosynthetic factories” to supply the building blocks for lipids, DNA and protein synthesis required to support cancer cell proliferation [4,5]. Mitochondria are unique organelles in many ways. Besides being the main site of cellular respiration and ATP production through oxidative phosphorylation (OXPHOS), they are crucial for fatty acid catabolism through the β-oxidative pathway, for anabolic metabolism of lipids, aminoacids and heme; they also participate in Ca^2+^ homeostasis, connect signaling pathways and apoptotic cascades. A tight coordination of nuclear and mitochondrial functions is required to maintain proper mitochondria functionality and to adjust mitochondrial activity to the energetic and biosynthetic requirements of the cell. A clear example of this coordination is the assembly of the respiratory complexes of the electron transport chain (ETC). Mitochondria have a circular DNA genome of 16.6 Kb that encodes for 13 subunits of complexes I, III, IV and V of the ETC, along with two ribosomal RNA and 22 mitochondria-specific t-RNA. The ETC complex assembly, therefore, requires a regulation of both nuclear-encoded and mitochondrial-encoded subunits, which need to be in proper stoichiometric ratios. Failure to maintain this proportion leads to the mito-nuclear protein imbalance, which could result in reduced mitochondrial respiration and ATP synthesis [6].

Mito-nuclear communications are exerted through the “anterograde signaling”, through which the nucleus regulates mitochondrial activity and number, and the “retrograde signaling”, which allows mitochondria to inform the nucleus about the onset of oxidative stress, ATP and metabolites levels, OXPHOS impairments, membrane potential disruption, accumulation of unfolded protein, thereby activating the proper nuclear transcriptional response [6,7]. It is becoming increasingly clear that transient and sub-lethal levels of mitochondrial oxidative stress elicit an adaptive response, termed “mitohormesis” that allows the cell to withstand more harmful stimuli, thus enhancing the cell resistance to apoptosis and prolonging lifespan [6,7,8]. Accumulating evidence is highlighting the importance of the mito-nuclear communication and mitohormesis in the onset and progression of metabolic, cardiovascular, neurological diseases, ageing and cancer.

Indeed, mitohormesis is a clear paradigm of the importance of mito-nuclear communications, since the stress-induced signaling originating from mitochondria elicit a nuclear response aimed at increasing the antioxidant defenses, to promote the mitochondrial turnover through mitophagy and biogenesis, and to remodel mitochondrial metabolism.

Remarkably, a transient increase in mitochondrial oxidative stress during fetal development triggers a stable hormetic response in the adult liver that heightens the basal level of mitochondrial antioxidant defense. This mitohormetic adaptive response requires the activation of nuclear respiratory factor (NRF2), peroxisome proliferator-activated receptors (PPARs) and the peroxisome proliferator-activated receptor gamma coactivator 1 (PGC1α) pathways [9]. While reactive oxygen species (ROS) are by far the more studied mitohormetic triggers, other stressors can elicit this adaptive response, working both together or independently of ROS, such as ions, metabolites, lipids, or nucleic acids [10,11,12]. Collectively, these signaling factors can act as “mitokines” promoting mitohormesis in an autocrine, paracrine and even endocrine manner [13].

As the main metabolic organ, in order to regulate the body energy metabolism, the liver needs to adapt its metabolic activity constantly, integrating different input signals such as nutrient and hormones levels, neuronal signaling, physical activity and circadian rhythms. Moreover, the liver is the main site of detoxification from xenobiotics and alcohol, which poses additional requirements to withstand oxidative and metabolic stressors. Despite the remarkable adaptive capacity of the liver, prolonged alcohol abuse, viral infections, genetic or metabolic disorders (non-alcoholic fatty liver disease, NAFLD and non-alcoholic steatohepatitis, NASH) can result in the exhaustion of the liver anti-oxidant defense, leading to chronic liver disease that can eventually progress to hepatocellular carcinoma (HCC). Hepatocellular carcinoma accounts for 75%–85% of total cases of primary liver cancer, it is currently the fifth most common cancer in men, the ninth in women and the second leading cause of cancer-related death worldwide [14]. The very high ratio of mortality to incidence (0.95) is indicative of the absence of effective therapeutic strategies, even if many treatment options have been developed during the last years, including hepatic resection, liver transplantation and molecular targeted therapies.

Metabolic reprogramming is a key event in hepatocellular carcinoma onset and progression [15]. Aberrant up-regulation of the mammalian target of rapamycin (mTOR) pathway occurs in up to 50% of HCC [16,17,18,19]. The mTOR pathway coordinates cellular metabolism and proliferation according to nutrient availability, to hormones and to growth factor signaling, by promoting the lipid, nucleotide and protein synthesis required for biomass growth. Increased de novo lipogenesis is a key metabolic reprogramming associated with HCC [20,21]. Activation of PI3K/AKT/mTOR signaling in HCC promotes lipogenesis, which exploits both mTORC1 and mTORC2 activation [22,23,24] and shuts-down lipid catabolism [25]. Importantly, the lipogenic program activated by mTOR through induction of sterol regulatory element-binding protein 1 (SREBP-1) is crucial for cell proliferation [26,27]. Indeed high expression of SREBP-1 correlates with increased cell proliferation of reduced survival in HCC patients [28].

Mitochondria defects are well documented in HCC. Many studies have identified frequent mutations of mtDNA [29], in particular in the D-loop [30,31,32,33,34], as well as reduced mtDNA content [34,35,36], resulting in dysfunctional mitochondria that are characterized by decreased OXPHOS [34,37] and increased ROS production [35,38]. Moreover, depletion of mtDNA in HCC has been correlated with increased resistance to pharmacological treatments [39,40,41]. A recent study by Li et al. highlighted the intra tumoral heterogeneity of mtDNA somatic mutations in Hepatitis B Virus (HBV)-related HCC. They found that HCC samples showed a higher degree of mtDNA mutations with respect to matched non-HCC tissues and that mtDNA mutations within HCC had a higher heteroplasmy than those in paratumoral tissue [42]. Moreover, all identified mtDNA mutations in theparatumoral samples were private (i.e., unique to that sample), whereas, on average, only ≈24% mtDNA mutations were private in HCC samples, suggesting a positive selection of mtDNA mutations in HCC. Interestingly, the burden of mtDNA somatic mutation in non-HCC samples of HBV-infected patients was found to be twice as much as that of healthy liver samples, suggesting that mtDNA may be a feature of HBV infection [42], although the pathogenic role of mtDNA mutations in HCC pathogenesis remains controversial [34,35,39,43,44]. Despite the growing number of studies identifying mitochondrial defects and mtDNA mutations in HCC, it is still debated if and to what extent these defects are positively selected and actively promote the progression of HCC or rather that merely reflect the higher oxidative damage and relaxed negative selection typical of tumors [34,35,39,43,44].

Nevertheless, active research is being conducted to disentangle the intricate connections between mito-nuclear communications and metabolic rewiring in HCC pathogenesis and progression. This review summarizes the recent advances in relevant aspects of the mitochondrial-nuclear communication, in the context of the metabolic reprogramming of hepatocellular carcinoma.

## 2. Anterograde Signaling

**Figure 1:**
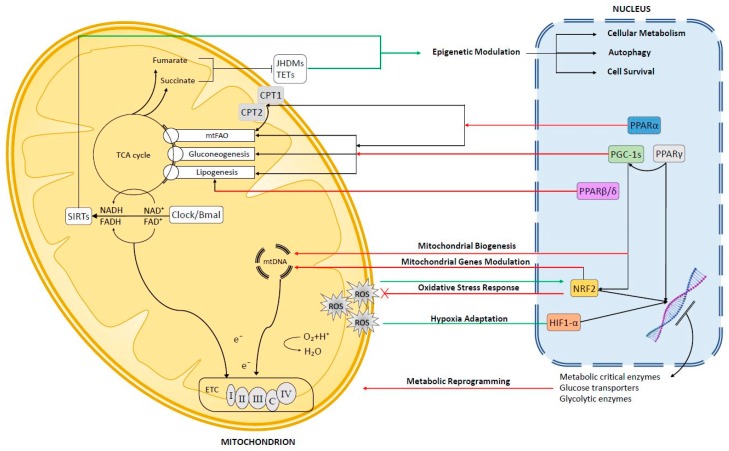
General overview of the mito-nuclear communication in liver cells. Anterograde signaling pathways highlighted in red, retrograde pathways in green.

Nuclear control of mitochondria is actuated mainly through the modulation of nuclear-encoded mitochondrial proteins, regulation of mitochondria translation, mitochondrial biogenesis, autophagy and dynamics. Nuclear receptors and transcription factors integrate intra- and extra-cellular signals, such as nutrient levels, hormones, stress signals, redox status, to drive the appropriate nuclear response. Within the nuclear receptor superfamily, PPARs and PGC-1s are well-known to play a key role in mitochondria biology (Figure 1).

### 2.1. Peroxisome Proliferator-Activated Receptors (PPARs)

PPARα, PPARβ/δ and PPARγ are the three members of the Peroxisome Proliferator Activated Receptors (PPARs) family. PPARs are potent regulators of glucose and lipid metabolism and the target of several synthetic drugs, such as fibrates (PPARα), TZD (PPARγ) and recently developed dual agonists (either PPARα-PPARβ/δ or PPARα-PPARγ).

#### 2.1.1. PPARα

In the liver PPARα is by far the most expressed PPAR isoform and is the master regulator of fatty acid (FA) disposal through the mitochondrial and peroxisomal β-oxidation (fatty acid oxidation, FAO), regulates ketogenic response and lipoprotein trafficking [45,46]. PPARα modulates mitochondrial metabolism by directly inducing the transcription of fatty acid transporters located in the outer (carnitine palmitoyltransferase 1, CPT-1) and inner (carnitine palmitoyltransferase 2, CPT-2) mitochondrial membrane [46,47], thereby facilitating FA translocation to the mitochondria. Here, FA are degraded through the β-oxidative pathway, a multi-step process that produces acetyl-CoA molecules from longer acyl-CoAs. The genes coding β-oxidative enzymes are direct target of PPARα transcriptional activity [46]. During fasting, PPARα promotes acetyl-CoA utilization in liver mitochondria as a substrate for the synthesis of ketone bodies, which are used in the tricarboxylic acid cycle (TCA) by peripheral tissues, via the induction of β-Hydroxy β-methylglutaryl-CoA Synthase (HMG-CoA) [48]. PPARα, as the other PPAR isoforms, induce the expression of mitochondrial uncoupling proteins (UCP1-3), which promote energy expenditure by futile FA oxidation [49]. The beneficial effect of the increased mitochondrial fatty acid disposal mediated by PPARα activation is well established in experimental models of NAFLD/NASH [50,51,52]. In humans, the dual PPARα-PPARβ/δ agonist Elafibranor was recently shown to ameliorate NASH in a subgroup of patients [53]. Increased disposal of FA decreases hepatocellular damage and inflammation, ameliorating the NASH phenotype.

Whether remodeling of mitochondrial metabolism by PPARα plays a beneficial or detrimental role in HCC is still debated. A protective role of PPARα in HCC development has been suggested in several experimental models. PPARα^−/−^ mice are more prone to diethylnitrosamine (DEN)-induced HCC than WT mice, due to the reduced activation of the pro-apoptotic Bcl2 cascade, mediated by nuclear factor kappa B, NF-kB [54]. PPARα prevents pyruvate entry in the mitochondria by PDK4-mediated inhibitory phosphorylation of pyruvate dehydrogenases [55], thus blocking glucose utilization in the TCA for energy production and lipid synthesis [56]. Anaplerosis from glutamine, by which cells replenish TCA intermediates that are consumed by biosynthetic processes, is commonly activated in cancer cells, including HCC [57], which may be completely dependent on glutamine for their growth [58,59]. PPARα represses the expression of glutaminase and glutamate dehydrogenase, thereby blocking anaplerosis from glutamine [55]. Importantly, actively proliferating hepatocytes, either HCC cells of normal hepatocytes after partial hepatectomy, were shown to suppress PPARα expression and FA β-oxidation through a mechanism regulated by CyclinD1. Suppression of CyclinD1 restored both PPARα expression and FAO, thereby directly linking hepatocyte proliferation to inhibition of PPARα-mediated β-oxidation [60]. These data are in accordance with the observation that, in human HCC samples, reduction of mitochondrial FAO due to downregulation of PPARα regulated genes such as hydroxyacyl-CoA dehydrogenase trifunctional multienzyme complex subunit alpha (HADHA) was shown to correlate with less differentiated cancers [61].

Liver lipid content is decreased by PPARα not only through FA disposal in the β-oxidative pathway, but also through the repression of lipid biosynthesis. PPARα induces the expression of MLYCD gene, which code for the malony-CoA degrading enzyme malonyl-CoA decarboxylase. Malonyl CoA is a precursor of FA biosynthesis and, in turn, prevents FA disposal by inhibiting the mitochondrial transporter CPT-1 [62]. The importance of this inhibitory regulation is highlighted by the very recent paper of Lally and colleagues, which elegantly showed how reducing lipogenesis by targeting the malonyl-CoA-carboxylase (the enzyme that convert acetyl-CoA into malonyl-CoA) effectively prevents HCC development in mice [63]. Moreover, point mutation in acetyl-CoA carboxylase 1 gene (ACC1) that prevents its inhibitory phosphorylation by AMPK, results in constitutive lipogenesis that enhance human HCC cell growth [63]. However, very recent work shows that in β-catenin activated HCC (Apc^hep−/−^ mice), PPARα-induced FAO is the driving force for energy production though OXPHOS and deletion of PPARα was sufficient to prevent HCC initiation and progression in the Apc^hep−/−^ model [64]. PPARα was shown to be a direct target of β-catenin (CTNNB1) in human HCC, and PPARα expression was higher in CTNNB1-mutated human HCC than in non-mutated tumors. Differently from other HCC molecular subtypes, such as AXIN1-mutated, β-catenin-activated HCC did not rely on lipogenesis for cell growth. On the contrary, Apc-HCC had a reduced acetyl-CoA flux into the lipogenic pathway, reduced levels of malonyl-CoA and reduced expression of lipogenic enzymes Acetyl-CoA carboxylase (Acac), fatty acid synthase (Fasn) and lipin1 (Lpin1) [64]. Therefore, different molecular subtypes of HCC may have divergent (even opposite) metabolic requirements for cell growth and precise characterization of their metabolism will be of crucial importance to develop effective therapeutic strategies.

#### 2.1.2. PPARβ/δ

In the liver, PPARβ/δ promotes glucose uptake and utilization (by inducing GLUT2, GK, pyruvate kinase) either to increase glycogen storage or to promote de novo lipogenesis (by inducing FAS, ACC1, ACC2, SCD1, SREBP-1c and PGC-1β), while coordinately prevents gluconeogenesis by inhibition of phosphoenolpyruvate carboxykinase (PEPCK) and hepatocyte nuclear factor alpha (HNF-4) [65]. PPARβ/δ was shown to be required for mitochondrial biogenesis and differentiation into hepatic-like tissue of mouse ES cells [66]. Indeed, transient induction of PPARα at the beginning of the differentiation process triggered PGC-1α induction activating mitochondrial biogenesis, while acquisition of terminal differentiation was dependent upon stable and sustained expression of PPARβ/δ, paralleled by the acquisition of high mitochondrial membrane potential and albumin expression [66]. In keeping with its role in hepatocyte differentiation, mice deleted of PPARβ/δ showed impaired liver regeneration after partial hepatectomy, lacked transient steatosis and induction of Akt and E2F signaling, which is associated with liver regeneration [67]. In particular, E2F factors are increasingly being recognized as coordinators of the glycolytic/oxidative metabolism switch, cell proliferation and apoptosis [68,69,70]. In HCC samples, PPARβ/δ expression was found reduced compared to adjacent non-tumoral tissue [71]. Moreover, by screening the expression of all nuclear receptors during liver regeneration, the authors found that PPARβ/δ expression was consistently associated with the non-proliferative status of hepatocytes. Pharmacological activation of PPARβ/δ in hepatoma cells reduced the expression of CyclinD1 and proliferation [71]. However, PPARβ/δ was recently described to be involved in the acquisition of resistance to sorafenib in HCC cells. The authors found that sorafenib-resistant cells acquired high glutamine metabolism and elevated PPARβ/δ expression. Glutamine anaplerosis was used to foster nucleotide synthesis through the Pentose-Phosphate-Pathway (PPP) and lipid biosynthesis [72], promoting cell proliferation and redox homeostasis. Increased expression of PPARβ/δ and Glutaminase, (GLS1) were detected in human HCC that acquired resistance to sorafenib, while pharmacological targeting of PPARβ/δ sensitized HCC cells to sorafenib in vitro and in xenograft models.

#### 2.1.3. PPARγ

PPARγ is the master regulator of lipogenesis and adipogenesis, but it is also a potent modulator of the inflammatory response, in particular in macrophages and endothelial cells. PPARγ promotes glucose uptake by inducing glucose transporters (GLUTs) and by modulating insulin sensitivity (IRS-1 and -2, PI3K) [73]. Glucose is then directed to the de novo lipogenesis pathway and triglyceride synthesis, by induction of acyl-CoA synthetase, glycerol kinase, PEPCK, among others [73,74]. Fatty acids uptake and mobilization are also modulated by PPARγ, through the regulation of transporters and lipases (FAT/CD36, fatty acids binding proteins aP2, lipoprotein-lipase). Importantly, PPARγ regulates whole-body glucose and lipid homeostasis by coordinate action on the liver, adipose tissue and muscle, which is mediated by a complex inter-organ communication network of circulating lipids, adipokines, hepatokines and inflammatory cytokines.

The role of PPARγ in HCC is still debated, as both pro- and anti- tumoral function were reported. As many studies were conducted in the past using PPARγ agonists (TZDs) which also elicit PPAR-independent anti-cancer effects [75,76], the interpretation of the experimental results in the literature is quite complicated. Indeed, we showed that TZD anti-tumoral effect on liver cancerogenesis in vivo was significantly higher in PPARγ^−/−^ mice that in wild-type ones [76].

Focusing on PPARγ-related function in mitochondria metabolism and HCC, it is worth noting that PPARγ is a bona fide target of the mTOR pathway [27], which is often deregulated in HCC [17]. Consistently, PPARγ is found to be induced in mTOR-driven HCCs [77]. Interestingly, in PTEN null mice PPARγ directly induce the expression of glycolytic gene hexokinaseand oncogenic pyruvate kinase M2 (PKM2), inducing hepatocyte steatosis, hypertrophy and hyperplasia [78]. This finding is consistent with the observation that the mitogenic MEK/ERK signaling promotes an activating phosphorylation of PPARγ at Ser84, resulting in the direct induction of glycolitic gene PFKFB4 and in HCC cell proliferation [79]. Alternative mechanism of activation of PPARγ, leading to increased glycolysis in HCC, are recently emerging and involve lcnRNAs and miRNAs [80,81]. Recently, novel PPARγ variants have been discovered which appear to directly modulate mitochondrial metabolism. A C-terminal truncated PPARγ2 isoform was found to stably associate with the D-loop of mtDNA in differentiating brown adipocytes. Forcing PPARγ2 localization in mitochondria, resulted in enhanced ETC gene expression and OXPHOS, showing that in brown adipocytes PPARγ2 can directly induce mtDNA expression [82]. Somewhat similarly, a recent study by Niu and collaborators shows that tumor -associated macrophage differentiation is promoted by caspase-1 cleavage of PPARγ. The cleaved PPARγ translocate to mitochondria, where it directly interacts with medium-chain acyl-CoA dehydrogenase (MCAD), thereby attenuating FAO and promoting lipid accumulation [83]. Of note, inhibition of the caspase-1/PPARγ/MCAD axis reduced tumor growth in a transgenic mouse model of breast cancer. Besides these new mechanisms, whose relevance in HCC still needs to be addressed, most of the established mitochondrial functions mediated by PPARγ are exerted through the recruitment of its coactivators PGC-1α and -1β, as discussed in the following section.

### 2.2. Peroxisome Proliferator Activated Receptor Co-Activators (PGC-1s)

Peroxisome proliferator activated receptor co-activators (PGC-1s), comprising PGC-1α, PGC-1β and PRC, are the main regulators of mitochondrial biogenesis, integrity and function. Modulation of virtually every aspect of mitochondrial biology is mediated by PGC-1s [84]. PGC-1s associate with several transcription factors (such as PPAR, ERR, NRF, GR, ER) and modulate their target specificity, coordinating the gene expression response in a cell-, tissue- and program- specific manner. Both PGC-1α and PGC-1β are potent inducers of mitochondria biogenesis; however, their functions in the liver seem to overlap only in part, since these PGC-1s are recruited by different pools of transcription factors or, alternatively, the same TF can be preferentially co-activated by PGC-1α or PGC-1β depending on different contexts. For example, PGC-1α co-activate the estrogen receptor and protects hepatocytes from the metabolic and oxidative damage of an obesogenic diet (HFD+30% Fructose in drinking water) [85] and in PGC-1α^−/−^ liver, compensatory increased of PGC-1β exerted a protective role. In a different experimental setting, estrogen effect on mitochondrial biogenesis seems to be selectively mediated by PGC-1β and not PGC-1α [86]. In the liver, PCG1α is induced by fasting, paralleling PPARα activation, and promotes gluconeogenesis, a process mediated by PPARβ/δ [87].

PGC-1s modulates the expression of the nuclear encoded ETC subunits and expression of mtDNA genes, by inducing the expression of NRF1 and NRF2 [88,89]. In turn, NRFs upregulates the expression of the nuclear-encoded mitochondrial transcription specificity factors (TFB1M and TFB2M) and TFAM, the latter being essential for transcription, replication and packaging of mtDNA [88,90,91,92]. Therefore, PGC-1α and -1β coordinate the expression of both nDNA and mtDNA encoded ETC proteins, directly regulation OXPHOS and mitochondrial biogenesis [93,94]. Importantly, PGC-1α and PGC-1β in the liver regulate metabolic functions that are largely divergent. PGC-1α co-activation of HNFα, forkhead box O1 (FOXO1), CAMP responsive element binding protein (CREB) and glucocorticoid receptors (GRs) is responsible for the induction of FA β-oxidation and gluconeogenesis [87,95], through transcriptional regulation of PEPCK and G6PD expression. However, it should be noted that PGC-1α has also been reported to induce genes involved in the de novo lipogenesis, such as ACC and FASN [96]. Conversely, PGC-1β is a poor activator of hepatic gluconeogenesis [95]; it is required for SREBP-1c induction of FA and cholesterol synthesis (through FASN and HMG-CoA Reductase, respectively), it is induced by dietary fatty acids and promotes lipoprotein secretion from the liver through activation of liver X receptor alpha (LXRα) [97]. Reflecting their divergent metabolic role, PGC-1α and -1β show antiphasic circadian regulation, with PGC-1α being upregulated at night and by fasting, while PGC-1β is induced by dietary FA intake [97] and show a diurnal rhythm [98]. Consistently, PGC-1β^−/−^ mice have greatly reduced activity during the dark cycle (mice are nocturnal animals and preferentially feed at night) [99]. Moreover, PGC-1α was shown to induce core clock genes, thus integrating metabolism and circadian regulation [100].

Given the key role of PGCs in metabolic reprogramming and mitochondrial homeostasis, several studies have focused on the role of PGCs in cancer development, including hepatocellular carcinoma. Whether PGC-1α acts as a tumor promoter or a tumor suppressor is highly debated, not only in HCC but also in several other cancer types [101,102]. Several lines of evidence support the role of PGC-1s in hepatocyte proliferation and HCC progression. Induction of PGC-1α is required to promote mitochondrial biogenesis and compensatory proliferation of hepatocytes surrounding the necrotic areas in the acetaminophen model of liver toxicity [103]. Mice PGC-1α^−/−^ were protected from DEN-induced HCC, as well as azoxymethane induced colon carcinogenesis [96]. Interestingly, in this paper the pro-tumoral effect of PGC-1α was found to be associated with the induction of lipogenic genes ACC and FASN [96]. PGC-1α was shown to mediate the adaptation of HCC cells to hypoxia by promoting mitochondrial biogenesis [104] and mitochondrial biogenesis activated by Sirtuin-1(SIRT1)/PGC-1α was found to foster EMT and HCC metastasis [105]. Activation of PGC-1α is well-known to promote HBV replication [106,107,108,109,110,111], thus possibly promoting HCC development.

Other studies have pointed out a tumor suppressor role of PGC-1α. Adenoviral-mediated expression of PGC-1α induced E-cadherin expression and reduced HepG2 migration [112], while in another study, overexpression of PGC-1α in the same HepG2 cell line was shown to induce apoptosis [113]. Silencing PGC-1α in L02 cells promoted a more de-differentiated phenotype, and PGC-1α was found to be down-regulated in human HCC samples [113]. In a mouse model of NASH-HCC (CDE diet), PGC-1α expression was reduced within the tumors, as well as in human HCC samples [114].The authors have shown that PGC-1α and other gluconeogenesis genes were reduced by miR-23a in experimental and human HCC, however, they did not investigate the effect of PGC-1α-targeting by miR-23a on mitochondrial biogenesis [114]. Interestingly, miR-23a is significantly up-regulated in human HCC vs. cirrhosis and its expression correlates with larger tumor size and progression [115]. Whether mitochondria biogenesis is regulated by miR-23a in hepatocellular carcinoma still needs to be defined. In other experimental settings, PGC-1α targeting by miR-23a was shown to impair mitochondrial function and promote mitochondria-mediated apoptosis [116,117,118]. Consistently, activation of AMPK-PGC-1α axis induces apoptosis of HCC cell lines [119].

Down-regulation of SIRT1 was shown to mediate the reduction in PGC-1α activity and consequent mitochondrial dysfunction in a model of glycogen storage disease 1a deficient in G6Pase-a, a progressive liver disease that can result in hepatocellular adenoma and hepatocellular carcinoma [120].

Inhibition of PGC-1α expression and reduction of gluconeogenesis was shown to be required for the tumor promoting activity of the Yes-associated protein 1 (YAP) in hepatocellular carcinoma. However, yes-associated protein 1 (YAP-1) repression of PGC-1α did not affect the expression of mitochondrial genes, suggesting that inhibition of gluconeogenesis, rather than remodeling mitochondrial function, promotes tumor growth by diverting substrates away from the energy-consuming processes of gluconeogenesis and toward anabolic pathways [121]. Interestingly, YAP was found to promote HCC cell migration by preventing JNK activation of Bnip3, a protein involved in excessive mitophagy, mitochondrial dysfunction and ATP shortage [122]. Mitochondrial dysfunction triggers intracellular calcium overload, activation of Ca^2+^/calmodulin-dependent protein kinases II (CaMKII) and inhibitory phosphorylation of cofilin, ultimately impairing F-actin polymerization and lamellipodium-based migration. Indeed, contrary to the glycolytic switch of primary tumor cells, PGC-1α mediated mitochondrial biogenesis and high OXPHOS seem to be a general requirement for metastatic cells [123].

Therefore, it seems that impairment of mitochondrial biogenesis and function could either promote or impair hepatocellular carcinoma development. The acquired resistance to the diverse stressors mediated by the mitohormetic response may contribute to the heterogeneity of response observed in HCC. Moreover, since telomere dysfunctions trigger a profound inhibition of mitochondrial biogenesis through p53-mediated suppression of both PGC-1α and PGC-1β [124], loss of function of this tumor suppressor adds a layer of complexity to the amount of stress that a cancer cell can adapt to before triggering mitochondria-mediated apoptosis.

The promoting role of PGC-1β in hepatocellular carcinoma is more consistent. In an elegant paper by Piccinin et al. the contribution of PGC-1β to hepatocarcinogenesis was recently highlighted. Overexpression of PGC-1β promoted hepatic carcinogenesis induced by DEN or by the genetic background (Abcb4^−/−^). Conversely, hepatocyte conditional PGC-1β^−/−^ mice were protected from DEN induced HCC [125]. The authors found that PGC-1β promoted the de novo lipogenesis and boosted the expression of mitochondrial ROS scavengers, thereby limiting oxidative stress-induced apoptosis of cancer cells [125]. As recalled above, increased de novo lipogenesis is a key metabolic reprogramming associated with HCC [20,21]. Interfering with de novo lipogenesis by pharmacologically mimicking the AMPK inhibitory phosphorylation of ACC1 and ACC2 effectively reduces DEN-induced HCC and the growth of HCC cells, in a cell-autonomous manner, in vitro and in vivo [63]. However, it should be noted that completely blocking de novo lipogenesis by ACC1/ACC2 deletion actually enhances DEN-induced HCC, by a mechanism likely dependent on the increased pool of NADPH and reduced glutathione, which improves survival to the oxidative damage of DEN [126].

Given the important extra-mitochondrial metabolic function of PGCs, it is not entirely clear to what extent their role on mitochondria is relevant to cancer cell oncogenic transformation, proliferation and chemoresistance. Indeed, metabolic adaptation (i.e., downregulation of gluconeogenesis and enhancement of lipid synthesis) could be a major driver in hepatocarcinogenesis, at least in part unrelated to mitochondria reprogramming. On the other hand, an hormetic response triggered by mitochondrial defects, such as loss or mutation of mtDNA caused by oxidative stress, would result in PGCs-mediated compensatory mitochondrial biogenesis. The up-regulation of PGCs required for the hormetic response could then, in principle, promote also their extra-mitochondrial functions. Supporting this scenario, PGC-1β was found to mediate the adaptive chemoresistance response associated with mtDNA mutations [93]. Both PGC-1α and PGC-1β were induced by cisplatin following mtDNA damage and mediated compensatory mitochondrial biogenesis in resistant cells; however, only PGC-1β was necessary for the acquired chemoresistance. Strikingly, the chemoresistance function of PGC-1β were found to be independent on the mitochondrial function of the co-activator [93].

A regulatory mechanism of PGC-1s activity that is relevant to hepatocarcinogenesis is their regulation by post-translational modifications, including phosphorylation by AMPK [127] and inhibitory acetylation. In particular, PGC-1α deacetylation by Sirtuins seems to play a role in several HCC-promoting mechanisms.

## 3. Mitochondrial Retrograde Signaling in Hepatocellular Carcinoma (HCC)

Under specific metabolic conditions, cells need to activate specific programs as an attempt to compensate for the on-going biological changes. In order to adjust to these conditions, cells can stimulate the transcription of nuclear target genes by mitochondrial retrograde signals. The retrograde communication can be triggered by fluctuations in metabolite levels, oxidative stress, energetic stress, and altered Ca^2+^ release.

As a result, epigenetic regulation programs, energetic and oxidative stress response in nucleus are activated to adapt cellular functions to the new metabolic requirements.

### 3.1. Reactive Oxygen Species (ROS)-Dependent Retrograde Signaling

Aerobic metabolism processes, like oxidative phosphorylation (OXPHOS) and ATP production, produce in mitochondria reactive oxygen species (ROS).

Although high levels of mitochondrial ROS are known to produce detrimental effects to the cell, it is now recognized that a controlled production of ROS plays a key role in regulating redox-sensitive proteins and activating downstream signaling pathways [6,128,129].

As recalled above, mtROS represent the main triggers that activate mito-nuclear communication in order to promote the mitohormetic response.

#### 3.1.1. Nuclear Factor Erythroid 2-Related Factor 2 (Nrf2)

Nuclear factor erythroid 2-related factor 2 (NRF2) is the best-known transcription factor regulating ROS-dependent retrograde signaling (Figure 1). Increased mitochondrial oxidative stress activates NRF2, which moves into the nucleus and binds consensus DNA sequences termed antioxidant response elements (AREs) on the promoter of target genes, thus increasing the transcription of detoxification and antioxidant enzymes [130,131,132,133]. Physiologically, Nrf2 is sequestered in the cytosol by his inhibitor Kelchlike ECH-associated protein 1 (Keap1) which regulates the availability of Nrf2 by acting as an adaptor for the CUL3/RBX1 E3 ubiquitin ligase complex, thereby mediating the rapid ubiquitination and proteasomal degradation of Nrf2 [134,135,136,137]. During redox-stress conditions, such as high mitochondrial ROS production, Keap1 is oxidized at redox sensitive cysteine residues and undergoes a conformational change that, ultimately, prevents Nrf2 ubiquitination and proteasomal degradation. The molecular details of the redox-sensitive Keap1-Nrf2 interaction under stress conditions are complex and the proposed models involve either the dissociation of the CUL3 complex from Keap1-Nrf2, or a cycling conformation “hinge and latch”, in which the Keap1 dimer interacts with Nrf2 with one monomer (open conformation) or with both monomers (closed conformation). In the closed conformation, the lysin residues of Nrf2 are not properly oriented for ubiquitination by the CUL3 complex, resulting in decreased Nrf2 degradation [138,139]. An evolution of this model proposes that the closed conformation impairs Keap1 recycling, leaving de novo synthetized Nrf2 free to accumulate and translocate into the nucleus [140].

Post-translational modifications such as phosphorylation by PKCδ [141] and AMPK [142] or acetylation by the CREB-binding protein acetylase promote the nuclear localization of NRF2 and its transcriptional activity, whereas deacetylation by SIRT1 increases NRF2 retention in the cytoplasm [143]. In the nucleus Nrf2 partner with small Maf proteins (sMaf) and the Nrf2-sMaf heterodimer then bind to ARE-containing promoters, thus activating the transcription of genes involved in antioxidant response and phase II detoxification enzymes such as NADPH quinone oxidoreductase (NQO-1), glutathione S-transferases (GSTs), heme oxygenase-1 (HMOX1), and glutamate-cysteine ligase catalytic subunits [144,145,146,147].

Similarly to other adaptive mechanism to stress, Nrf2 shows a dual role in the onset and progression of cancer [148]: on one hand it suppresses the malignant transformation by protecting cells from oxidative damage [149], on the other cancer cells can exploit the same mechanism to adapt and proliferate in the harsh tumor microenvironment [150,151,152]. Oxidative stress is a key determinant in the development of HCC causing DNA damage, accumulation of protein adducts, membrane lipo-peroxidation and multi-organelle damage which further increases ROS production [129,153,154].

The expression levels of Nrf2 in end-stage liver disease and HCC has been discrepantly reported. NRF2 mRNA expression was reduced in HCC tissues compared to matched non-tumoral samples while KEAP1 expression was generally conserved, leading to a decreased NRF2/KEAP1 ratio [147,155]. Chen and coworkers reported an increased level of phosphorylated and not-phosphorylated Nrf2 protein in a larger series of HCC after curative resection. Patients with higher nrf2 and lower keap1 expression were found to have a significantly reduced overall survival(OS) and disease-free survival(DFS) [156], Accordingly, Zhang et al. found an increased Nrf2 protein expression in a series of 65 HCC samples, where Nrf2 expression positively correlated with metastasis at distal sites and lower OS and DFS. In vitro, they found that Nrf2 expression promoted proliferation and invasion of HCC cell lines [157]. These conflicting reports may reflect the differences in mRNA vs. protein analysis, considering that Nrf2 is tightly regulated at the post-translational level, as well as the heterogeneity of HCC samples.

Interestingly, the Nrf2 target gene NQO-1 directly interacts with hypoxia inducible factor 1 subunit alpha (HIF-1α) and inhibits its degradation [158], suggesting a potential mechanism for the increased angiogenesis and malignity observed in some NRF2 positive tumors [159,160].

The activation of the NRF2/KEAP1/ARE transcriptional pathway plays a crucial role in glycolytic metabolic switch, increasing the glucose utilization as principal energy source. It has been reported that NRF2 indirectly induces G6PD expression by down-regulating miR-1 [161]. In several human HCC there is a significant upregulation of G6PD. Microarray analysis of HCC biopsies confirm an increased G6PDH expression in association with a reduction of miR-1 expression levels. Moreover, in vitro studies demonstrate that NRF2-silenced HCC cells down-modulate hexokinase 2 (HK II), citrate synthase (CS), TNF receptor associated protein 1 (TRAP1) and HIF-1α, further indicating a central function of NRF2 as in metabolic rewiring [162].

#### 3.1.2. Hypoxia-Inducible Transcription Factor (Hif1-α)

One of the most important features that characterizes the cancer microenvironment is low O_2_ levels environment (i.e., hypoxia) [163]. In normally oxygenated tissues, O_2_ levels ranges somewhat from 4 to 7.5%, while in tumors, due to fast growing rates and poor vasculature supply, O_2_ levels drop around and often below 1% [164]. Despite being an highly vascularized organ, hypoxia can occur in the liver as a result of the tissue remodeling caused by fibrosis and cirrhosis and HCC are reported to be among the more hypoxic tumors even though are able to potently induce neoangiogenesis [165]. As normal cells rely on oxygen for energy production by OXPHOS, they have evolved conserved mechanism to adapt to the hypoxic environment by extensively remodeling their energetic metabolism. Hypoxia-inducible transcription factors (HIFs) are oxygen sensitive transcription factors that play a key role in this adaptive response [166]. HIF heterodimers consist of HIF-1α and HIF-1β subunits; despite both are constitutively expressed under physiological O2 levels HIF-1α undergoes a quickly ubiquitination-dependent proteasomal degradation [167]. The targeted degradation of HIF-1α is a two-step process that requires an initial hydroxylation by the α-ketoglutarate-dependent prolyl hydroxylase 2 (PHD2) and subsequent polyubiquitination by the von Hippel-Lindau (VHL) ubiquitin ligase [168]. Hypoxia inhibits PHD2 activity, resulting in reduced degradation and stabilization of HIF-1α. Stabilized HIF-1α accumulates and translocates into the nucleus, where it dimerizes with HIF-1β. HIF-1 dimers binds to Hypoxia Response Elements (HREs) in the promoters of target genes, activating the hypoxic response (Figure 1) [167].

The transcriptional response to hypoxia regulated by HIFs activate genes involved in angiogenesis and O_2_ supply, cell proliferation, stemness, EMT, apoptosis and resistance to apoptosis, invasion and metastasis [169]. One of the larger cluster of genes regulated by HIF-1α is related to the induction of glycolytic genes and glucose utilization and repression of oxygen consuming processes such as OXPHOS [169].

Many cancer types, including HCC, exploit HIF-1α-mediated metabolic reprogramming independently of hypoxia. Of note, HIF-1α is a transcriptional target of the mTORC1 complex [27] while, under hypoxic conditions, HIF-1α inhibit mTOR signaling to reduce oxygen utilization [170].

Indeed, cancer cells can activate a pseudo-hypoxic response, mediated by the reduction of α-ketoglutarate and the accumulation of succinate which occurs, for instance, in succinate dehydrogenase-mutated cells. Succinate is the end-product formed by PHD during the α-ketoglutarate-dependent hydroxylation of HIF-1α, and as such, inhibits PHD activity [171]. Therefore, accumulation of succinate (and to lesser extent fumarate) in mitochondria leads to HIF-1α stabilization independently of hypoxia [172].

In human HCC samples, high levels of HIF-1α protein are associated with poor prognosis [173,174,175]. Moreover, HIF-1α activation of glycolysis was found to significantly correlate with a more aggressive behavior of HCC [176,177,178]. As for several other cancer types, HIF-1α promote the resistance to drug-induced apoptosis chemoresistance of HCC cells [179,180,181,182,183,184,185].

Mitochondrial ROS have a main role HIF stabilization [186]. Indeed, many studies report an inability of cells lacking mitochondrial DNA (ρ° cells) to stabilize HIF-1α subunit in hypoxic conditions [187,188].

Since ρ° cells are depleted of mtDNA, they are unable to produce key ETC proteins and are therefore highly defective in OXPHOS, resulting in very low mtROS production [188]. Accordingly, mitochondria-replete cells fail to stabilize HIF-1α under hypoxia if treated with OXPHOS inhibitors [189]. Restoring the mtDNA content in ρ° cells rescue OXPHOS, mtROS production and HIF-1α stabilization under hypoxia, further substantiating the essential role of mtROS for HIF-1α activation [187]. Indeed, mitochondria could maximize mtROS production from complex III in order to satisfy cell requests under hypoxic conditions [190]. mtROS generated specifically at complex III are required for HIF activation, as suggested in several studies that show a failure of HIF-1α stabilization when cells lose their ability to generate mtROS from complex III [191,192,193,194,195]. Of importance, HIF-1α stabilization by complex III-derived ROS does not require OXPHOS [191,193]. Mechanistically, mtROS were shown to stabilize HIF-1α through the inhibition of PHD2 enzymatic activity [195,196,197].

### 3.2. NAD^+^-Dependent Retrograde Signaling

Sirtuins (SIRT-1 to -7) are a family of class III NAD^+^-dependent histone deacetylases (HDAC) homolog to the yeast Sir2. SIRTs are able to deacetylate non-histone targets, including several transcription factors and signaling proteins. Members of the SIRT family are localized in different organelles: SIRT-3, -4, and -5 are found in the mitochondrial matrix [198], SIRT-6 and -7 are nuclear proteins, SIRT-1 and SIRT-2 are mainly nuclear and cytoplasmic, respectively, but able to shuttle between the two compartments [199] and, at least for SIRT1, possibly also to mitochondria [200]. As the deacetylation activity of sirtuins depends on NAD^+^ their activity is intrinsically linked to mitochondrial metabolism and NAD^+^/NADH ratio (Figure 1). NADH is produced by glycolysis and TCA cycle, while NAD^+^ is regenerated through oxidation of NADH by the ETC complex I (NADH dehydrogenase), through oxidation by lactate dehydrogenase (LDH) in glycolytic cells, de novo synthetized from precursor (tryptophan or nicotinic acid) or recycled through salvage pathways [201]. Cytosolic and mitochondrial NAD Kinases convert NAD^+^ to NADP^+^ which is essential (in the reduced form NADPH) for anabolic reactions, detoxifications and mitochondrial antioxidant defenses [202].

SIRT1 is able to deacetylate a plethora of non-histone targets, many of which are key regulators of cellular metabolism (PPARγ, SREBP1c, FXR, LXR, FOXO1, AMPK) mitochondrial biogenesis (PGC-1α), autophagy (Atg5, Atg7, Atg8/LC3), circadian clock (CLOCK, BMAL, PER2) and cell fate (p53) [201,203,204]. PGC-1α transcriptional activity is tightly controlled by post-translational modifications, including phosphorylation and acetylation. Under high-nutrient conditions and low NAD^+^ levels, General control of amino acid synthesis 5 (GCN5), represses PGC-1α by acetylation on multiple lysine residues and sequestering it in punctate nuclear speckles [205] (the same occurs for PGC-1β [206]). Upon-fasting, NAD^+^ levels increase, promoting SIRT1 activity and deacetylation of PGC-1α, allowing its full transcriptional activity [207].

SIRT1 is expressed at very low levels in normal liver, but it is overexpressed in HCC cell lines and in a subset of HCC, where its expression correlates with tumor stage [208,209]. However, SIRT1 was shown to act as a tumor suppressor in a large series HBV-related p53-mutated HCC [210]. Activated (phosphorylated) SIRT1 was a prognostic factor for longer relapse-free survival in p53-mutated tumors and significantly correlated with active AMPK. In vitro, the authors showed that SIRT1 is required for the activation of AMPK and consequent inhibition of mTOR signaling in p53-mutated HCC cells, resulting in growth arrest. Moreover, metformin (an activator of AMPK), was specifically effective in reducing the growth of tumors with mutant p53 and inactive SIRT1. These data suggest that the pro- vs. anti–oncogenic functions of SIRT1 depends on p53 mutation status, although the molecular details of SIRT1-AMPK-p53 interaction are not clear and require further investigations [210].

In the liver, SIRT-1 also deacetylates mitofusin-2 (but not MFN-1) [211], a major regulator of mitochondrial shape and fission/fusion dynamics, thus allowing efficient mitophagy and protecting from I/R injury [211,212]. However, in HCC mitochondrial remodeling by mitofusin-2 seems to produce a rather different outcome. Indeed, overexpression of mitofusin-2 reduces mitochondrial fission and triggers Ca^2+^ release, activating the Bax/Cytochrome-c mediated apoptotic program [213,214]. Mitofusin-2 expression was also found downregulated in HCCs samples respect to adjacent non-tumor tissue [213,215,216]. Down-regulation of MFN-2 correlated with disease progression and worse survival. Interestingly, gene expression profiling revealed that focal adhesion and PI3K-AKT pathway were significantly related to MFN-mediate signaling [215]. Disruption of mitochondrial dynamics toward fission and mitophagy is operated by HBV to promote cell survival and viral persistence. HBV induces dynamin-1-like protein (drp1) translocation to mitochondria and Parkin-mediated degradation of mitofusin-2, thereby promoting mitochondrial fission [217]. Consistently, the major regulator of mitochondrial fission dpr-1, was found significantly associated with distant metastasis in human HCCs, while mitofusin-1 showed an opposite trend [218]. Mechanistically, mitochondrial fission promotes lamellipodia-mediated migration of HCC cells through typical Ca^2+^/CaMKII/ERK/FAK pathway [218]. Since Drp1 is transcriptionally regulated by p53 [219], which is inhibited by SIRT1, it is tempting to speculate that mitochondrial dynamics and mitophagy may be regulated by SIRT1 depending on the mutation status of p53.

The expression of the mitochondrial SIRT3 is consistently reported to be downregulated in HCC samples, where its decreased expression correlates with reduced overall survival, tumor progression and recurrence [220,221,222,223,224,225]. Mechanistically, several lines of evidence support the tumor-suppressor activity of SIRT3 in HCC. First, SIRT3 promote the mitochondrial translocation of Bax via activation of the glycogen synthase kinase 5 beta (GSK-5b) pathway thereby promoting apoptosis [221]. Secondly, SIRT3 expression correlates with superoxide dismutase 2 (SOD2), a major mitochondrial ROS scavenger, pointing towards a protective role of this sirtuin from oxidative damage [226]. Interestingly, Ca2+ uptake into mitochondria inhibited the SIRT3/SOD2 pathway and activated JNK/MMP2, promoting cancer cell invasion and metastasis [226]. Moreover, loss of SIRT3 is involved in HCC chemoresistance to sorafenib and other chemotherapeutic agents. Mechanistically, SIRT3 downregulates the expression of Glutathione S-transferase pi 1 (GSTP1), an enzyme involved in cellular detoxification and drug resistance [227]. Therefore, loss of SIRT3 promotes HCC survival and resistance to treatments. Intriguingly, SIRT3 knockout (KO) mice were shown to have defective β-oxidation under fasting, calories restriction or cold exposure, due to hyperacetylation of acetyl-coenzyme A synthetase, long-chain acyl-coenzyme A (acyl-CoA) dehydrogenase (LCAD), and 3-hydroxy-3-methylglutaryl CoA synthase 2 [228,229]. In these mice, HFD accelerated the onset of obesity, insulin resistance and hyperlipidemia due to hyperactivation of the lipogenic enzyme stearoyl-CoA desaturase 1 [230], thus reinforcing again the link between mitochondrial fatty acid metabolism and HCC development.

Recently, the tumor suppressor role in HCC of another mitochondrial sirtuin was highlighted. Decreased SIRT4 expression in HCC patients correlates with shorter disease-free survival, and its deficiency promoted HCC lung metastasis in xenograft and DEN-treated SIRT4 KO mice [231]. SIRT4 does not have a strong deacetylase activity, rather, it acts as ADP/ribosyltransferase under nutrient-rich conditions, inhibiting glutamine catabolism by repressing glutamate dehydrogenase, thus preventing glutamine entry into the TCA cycle [59,232]. Wang and collaborators found that loss of SIRT4 promotes glutamine utilization for mitochondrial energy production by HCC cells, in accordance with the known function of SIRT4 [232]. Moreover, they found that loss of SIRT4 promoted the activation of mTOR pathways and inhibition of AMPK activity, while overexpressing SIRT4 elicited the opposite effect, substantiating a loop of reciprocal regulation between SIRT4 and mTOR pathway [59,231].

SIRT5 is involved in the regulation of multiple post-translational lysine modifications, including acetylation, succinylation, malonylation, and glutarylation [233,234,235]. Analysis of the succinylated proteome in SIRT5 KO mice liver revealed that although the majority of the proteins localized to mitochondria, a significant proportion were cytoplasmic and also nuclear [234]. Aminoacid catabolism, TCA and fatty acids metabolism are among the metabolic processes known to be regulated by SIRT5 in mitochondria. Recently accumulating evidences point towards a role of SIRT5 in HCC, although both pro- and anti- tumoral effect are being reported, along with several non-mitochondrial targets of this sirtuin. SIRT5 mRNA was found overexpressed in HCC samples compared to adjacent non-tumoral tissue and its expression correlated with tumor size, lymph node metastasis and TNM stage [236]. In vitro, down-regulation of SIRT5 decreased cell proliferation and invasion in HCC cell lines. The authors found that the pro-proliferative and migratory effects of SIRT5 were mediated, at least in part, by induction of E2F1. Direct binding of SIRT5 to the E2F1 promoter highlights extra-mitochondrial mechanism of action of SIRT5 in HCC [236]. Accordingly, SIRT5 was shown to be a direct target of miR-229-3p, which lower expression in HCC correlated with disease progression and poor survival. Mechanistically, miR-229-3p reduced HCC cell migration, invasion and proliferation through the downregulation of SIRT5, as shown by rescue experiments [237]. In another study, SIRT5 expression was found significantly downregulated in the LIHC cohort of the Cancer Genome Atlas (RNA-seq data) and in a small series of HCC and paired adjacent non-tumoral tissue (protein expression data) [238]. In this work, SIRT5 was found to suppress EMT in HCC cell lines and to inhibit cell migration by directly deacetylating vimentin at K120. The involvement of SIRT5 in another extra-mitochondrial pathway relevant to HCC has been recently shown by Chen and collaborators. They found that acyl-CoA oxidase1 (ACOX1), the rate-limiting enzyme of peroxysomal fatty acid β-oxidation, is suppressed by desuccinylation by peroxisomal SIRT5, thereby reducing H_2_O_2_ production and DNA oxidative stress damage [239]. Reduced expression of SIRT5 (protein data) was found in 78 paired HCC samples, with respect to adjacent normal tissue. In tissue microarray of 316 HCC samples the authors could establish that reduced SIRT5 expression correlated with worse overall survival and increased recurrence of HCC. Finally, SIRT5 protein expression negatively correlated with the DNA damage marker histone H2AX in a separate cohort of 116 HCC samples, further substantiating its protective role with respect to DNA oxidative damage in HCC [239].

The different pro- vs. anti- cancer functions of mitochondrial sirtuins may be possibly interpreted on the basis of the recently defined sirtuin interactome by the seminal work of Yang and colleagues. By using systematic proteomic approach, they were able to build a high-confidence network of protein interactions between SIRT-3, -4, -5 and proteins with validated mitochondrial localization [240]. The emerging picture is that each sirtuin interact with distinct clusters of mitochondrial proteins, suggesting non-redundant roles for these sirtuins. However, the partial-overlapping interactome of SIRT-3 and SIRT-4 suggest that these proteins may work in concert regulating common partners, although through separate physical association, while SIRT5 mitochondrial interactome, which is the smallest, appear to be clearly divergent from SIRT-3 and-4 [240].

### 3.3. Mitochondrial Metabolism and Epigenetic Regulation in HCC

In recent years several studies have suggested that epigenetic changes and alterations may be the main driving mechanisms of HCC development and promotion. These modifications cause a regulation of oncogenes and tumor suppressor genes [241,242,243,244,245]. The abrogation of metabolic pathways represents the most likely way to induce epigenetic modifications in cancer [246,247,248].

This is a common scenario of HCC development in which dysregulated mitochondria lead to an abnormal metabolites production, such as fumarate and succinate [249]. In a tumoral context they can act as “oncometabolites”, because the reduced turnover or changes to synthesis of these metabolites could perform an epigenetic control on nuclear gene expression generally through histone acetylation/deacetylation and DNA methylation/demethylation competing with nuclear enzymes [246]. This epigenetic control results in the modulation of genes involved in HCC progression, such as Ras association domain family member 1 (RASSF1), GATA binding protein 4 (GATA4), and cyclin dependent kinase like 2 (CDKL2) [242,250].

Many human cancers, including HCC, show defects of succinate dehydrogenase (SDH) and fumarate dehydrogenase (FH) [249]. Consequently, the loss-of-function of these enzymes cause an accumulation of succinate and fumarate. Furthermore, inhibition of SDH has been found to improve the chemosensivity of HCC cells [251]. SDH is a highly conserved heterotetrameric protein (composed by SDHA and SDHB as catalytic subunits, and SDHC/SDHD as structural subunits) encoded in the nucleus and then translocated to the mitochondrial inner membrane. This important mitochondrial enzyme of the TCA cycle catalyzes the oxidation of succinate to fumarate with the simultaneous reduction of ubiquinone to ubiquinol in the electron transport chain [252]. High concentrations of succinate and fumarate are able to inhibit α-ketoglutarate-dependent dioxygenases, like the Jumonji-C histone demethylases (JHDMs) and the Ten-eleven translocation methylcytosine dioxygenase (TET) family of 5-methylcytosine hydroxylases, resulting in genome-wide alterations of histone and DNA methylation and epigenetic dysregulation (Figure 1) [253,254,255,256,257,258]. TET is a three-member family (TET1, TET2, TET3) and catalyzes the conversion of the modified DNA base 5-metylcytosine (5-mC) to 5-hydroxymethylcytosine (5-hmC) [259]. TET proteins convert 5-mC to 5-hmC by oxidation of 5-mC in a Fe(II) and α-KG-dependent manner [66,259]. The 5-mC oxidative pathway mediated by the TET proteins may be relevant for activation or repression of gene expression by associating with transcriptional repressors or activation factors [259,260,261].

In the last years altered 5-hmC has been reported in different types of cancers playing an important role in the pathogenesis of many cancers, including HCC [262,263,264,265,266]. A recent study demonstrated a significant reduction of 5-hmC concentration in HCC tissues compared to non-tumor tissues. The decreased level of 5-hmC in HCC positively correlated with tumor size, AFP level and reduced overall survival, while a decreased level in non-tumor tissues was a prognostic factor the for early recurrence of HCC after surgical resection [266]. In parallel, increasing levels of 5-mC (corresponding to decreased level of 5-hmC) were detected in HCC tissues and significantly correlated with capsular invasion, vascular thrombosis, tumor recurrence and reduced overall survival. During DEN-induced liver carcinogenesis in rats, 5-hmC levels progressively decreased during cancer induction and further dropped in upon HCC development suggesting that 5-hmC is a critical actor in hepatocarcinogenesis. Furthermore, TET1 (but not TET2 or TET3) protein expression was found decreased in HCC samples respect to matched non-tumoral tissue, indicating that this TET1 may mediate the 5-mC/5-hmC unbalance in HCC [266].

## 4. Conclusions

For a long time, mitochondria have been mainly regarded as the cell “power-house”. However, it is now clear that these unique organelles are much more than that. The deep integration of mitochondria in every aspect of the cell regulatory network is rapidly emerging, revealing an unforeseen complexity of interactions between energy metabolism, stress-response, survival and apoptotic pathways, epigenetic regulation, circadian rhythm. Therefore, disruption of the mitochondrial communication network is a key event in many human diseases, including aging, cancer, immune response and, of course, metabolic disease.

Given the complexity of these regulations, the employment of unbiased, multi-omics approaches will be extremely valuable to disentangle the mitochondrial interactome with the nucleus and other organelles. Moreover, since HCC often occurs in the context of metabolic diseases, it will be crucial to address the alteration of hepatic mito-nuclear communication in the light of the whole-body metabolic dysfunction, integrating data from experimental models and patients [267,268].

As we gain a more robust understanding of the mitochondria as a communication hub, new therapeutic opportunities will hopefully begin to emerge also for those disease, such as HCC, that have currently very limited curative options.

Within the cell, we may say, all roads lead to mitochondria.

## Abbreviations

OXPHOS	Oxidative phosphorylation system
NAFLD	Non-alcoholic fatty liver disease
NASH	Non-alcoholic steatohepatitis
HCC	Hepatocellular carcinoma
mTOR	Mammalian target of rapamycin
PI3K	Phosphoinositide 3-kinase
AKT	Protein kinase B
mTORC1	Mammalian target of rapamycin complex 1
mTORC2	Mammalian target of rapamycin complex 2
SREBP-1	Sterol regulatory element-binding protein 1
PPARs	Peroxisome Proliferator Activated Receptor
PGC-1s	Peroxisome proliferator-activated receptor gamma coactivator 1
TZD	Thiazolidinedione
FA	Fatty acid
FAO	Fatty acid oxidation
CPT-1	Carnitine Palmitoyltransferase 1
CPT-2	Carnitine Palmitoyltransferase 2
TCA	Tricarboxylic acid cycle
HMG-CoA	β-Hydroxy β-methylglutaryl-CoA
UCP	Uncoupling Protein
DEN	Diethylnitrosamine
NF-kB	Nuclear Factor Kappa B
HADHA	Hydroxyacyl-CoA Dehydrogenase Trifunctional Multienzyme Complex Subunit Alpha
MLYCD	Malonyl-CoA Decarboxylase
ACC1	Acetyl-CoA carboxylase 1
AMPK	5′ adenosine monophosphate-activated protein kinase
CTNNB1	Catenin Beta 1
ACAC	Acetyl-CoA Carboxylase
GLUT2	Glucose transporter 2
GK	Glycerol Kinase
FASN	Fatty acid synthase
ACC2	Acetyl-CoA carboxylase 2
SCD1	Stearoyl-CoA Desaturase 1
PEPCK	Phosphoenolpyruvate carboxykinase
FASN	Fatty Acid Synthase
HNF-4	Hepatocyte Nuclear Factor 4 Alpha
PPP	Pentose-Phosphate-Pathway
GLS1	Glutaminase
PTEN	Phosphatase and Tensin Homolog
HK	Hexokinase
PKM2	Pyruvate Kinase M2
MEK	Mitogen-activated Protein Kinase Kinase
ERK	Extracellular signal–regulated Kinases
PFKFB4	6-Phosphofructo-2-Kinase/Fructose-2,6-Biphosphatase 4
MCAD	Medium-chain acyl-CoA dehydrogenase
ERR	Estrogen Related Receptors
NRF	Nuclear Respiratory Factor
GR	Glucocorticoid receptor
ER	Estrogen Receptor
TFB1M	Transcription Factor B1, Mitochondrial
TFB2M	Transcription Factor B2, Mitochondrial
TFAM	Transcription Factor A, Mitochondrial
HNFα	Hepatocyte Nuclear Factor Alpha
FOXO1	Forkhead Box O1
CREB	CAMP Responsive Element Binding Protein
PEPCK	Phosphoenolpyruvate Carboxykinase
G6PD	Glucose-6-Phosphate Dehydrogenase
LXRα	Liver X Receptor Alpha
SIRT1	Sirtuin 1
YAP	Yes-associated protein 1
JNK	c-Jun N-terminal Kinases
BNIP3	BCL2/adenovirus E1B 19 kDa protein-interacting protein 3
CaMKII	Ca2+/calmodulin-dependent protein kinases II
NRF2	Nuclear factor erythroid 2 related factor 2
AREs	Antioxidant response elements
KEAP1	Kelchlike ECH-associated protein 1
CUL3	Cullin 3
RBX1	Ring-Box 1
PKCδ	Protein kinase C delta
sMAF	small Maf proteins
NQO-1	NADPH quinone oxidoreductase
GSTs	Glutathione S-transferases
HMOX1	Heme oxygenase-1
HK II	Hexokinase 2
CS	Citrate Synthase
TRAP1	TNF Receptor Associated Protein 1
HIF-1α	Hypoxia Inducible Factor 1 Subunit Alpha
HIFs	Hypoxia Inducible Factors
HIF-1β	Hypoxia Inducible Factor 1 Subunit Beta
PHD2	α-ketoglutarate-dependent prolyl hydroxylase 2 PHD2
VHL	von Hippel-Lindau ubiquitin ligase
HREs	Hypoxia Response Elements
HDAC	NAD+-dependent histone deacetylases
LDH	Lactate dehydrogenase
FXR	Farnesoid X Receptor
CLOCK	Clock Circadian Regulator
BMAL	Brain and Muscle ARNT-Like
PER2	Period Circadian Regulator 2
GCN5	General control of amino acid synthesis 5
MFN-1	Mitofusin 1
MFN-2	Mitofusin 2
FAK	Focal Adhesion Kinase
Drp1	Dynamin-1-like Protein
GSK-5b	Glycogen Synthase Kinase 5 Beta
SOD2	Superoxide Dismutase 2
MMP2	Matrix Metallopeptidase 2
GSTP1	Glutathione S-transferase pi 1
LCAD	Long-chain acyl-coenzyme A (acyl-CoA) dehydrogenase
LIHC	Liver Hepatocellular Carcinoma
RASSF1	Ras Association Domain Family Member 1
GATA4	GATA Binding Protein 4
CDKL2	Cyclin Dependent Kinase Like 2
FH	Fumarate dehydrogenase
SDH	Succinate dehydrogenase
JHDMs	Jumonji-C histone demethylases
TET	Ten-eleven translocation methylcytosine dioxygenase
